# Magnesium–ibogaine therapy in veterans with traumatic brain injuries

**DOI:** 10.1038/s41591-023-02705-w

**Published:** 2024-01-05

**Authors:** Kirsten N. Cherian, Jackob N. Keynan, Lauren Anker, Afik Faerman, Randi E. Brown, Ahmed Shamma, Or Keynan, John P. Coetzee, Jean-Marie Batail, Angela Phillips, Nicholas J. Bassano, Gregory L. Sahlem, Jose Inzunza, Trevor Millar, Jonathan Dickinson, C. E. Rolle, Jennifer Keller, Maheen Adamson, Ian H. Kratter, Nolan R. Williams

**Affiliations:** 1 Brain Stimulation Lab, Department of Psychiatry & Behavioral Sciences, Stanford School of Medicine, Stanford, CA USA; 2 Palo Alto University, Palo Alto, CA USA; 3 Polytrauma Division, Veterans Affairs Palo Alto Health Care System, Palo Alto, CA USA; 4 Ambio Life Sciences, Vancouver, British Columbia Canada; 5 WRIISC-WOMEN & Department of Rehabilitation, Veterans Affairs Palo Alto Health Care System, Palo Alto, CA USA; 6 Department of Neurosurgery, Stanford School of Medicine, Stanford, CA USA; 7 Department of Radiology, Stanford School of Medicine, Stanford, CA USA

**Keywords:** Outcomes research, Psychology

## Abstract

Traumatic brain injury (TBI) is a leading cause of disability. Sequelae can include functional impairments and psychiatric syndromes such as post-traumatic stress disorder (PTSD), depression and anxiety. Special Operations Forces (SOF) veterans (SOVs) may be at an elevated risk for these complications, leading some to seek underexplored treatment alternatives such as the oneirogen ibogaine, a plant-derived compound known to interact with multiple neurotransmitter systems that has been studied primarily as a treatment for substance use disorders. Ibogaine has been associated with instances of fatal cardiac arrhythmia, but coadministration of magnesium may mitigate this concern. In the present study, we report a prospective observational study of the Magnesium–Ibogaine: the Stanford Traumatic Injury to the CNS protocol (MISTIC), provided together with complementary treatment modalities, in 30 male SOVs with predominantly mild TBI. We assessed changes in the World Health Organization Disability Assessment Schedule from baseline to immediately (primary outcome) and 1 month (secondary outcome) after treatment. Additional secondary outcomes included changes in PTSD (Clinician-Administered PTSD Scale for DSM-5), depression (Montgomery–Åsberg Depression Rating Scale) and anxiety (Hamilton Anxiety Rating Scale). MISTIC resulted in significant improvements in functioning both immediately (*P*_corrected_ < 0.001, Cohen’s *d* = 0.74) and 1 month (*P*_corrected_ < 0.001, *d* = 2.20) after treatment and in PTSD (*P*_corrected_ < 0.001, *d* = 2.54), depression (*P*_corrected_ < 0.001, *d* = 2.80) and anxiety (*P*_corrected_ < 0.001, *d* = 2.13) at 1 month after treatment. There were no unexpected or serious adverse events. Controlled clinical trials to assess safety and efficacy are needed to validate these initial open-label findings. ClinicalTrials.gov registration: NCT04313712.

## Abstract (web summary)

Military veterans with traumatic brain injuries who received treatment with the psychoactive compound ibogaine and magnesium experienced improvements in disability, psychiatric symptoms and cognition.

## Main

TBI is a leading cause of injury-related disability worldwide and is likely to remain so until at least 2030 (ref. ^1^). It is also the signature injury of US veterans from recent military conflicts, most often caused by blast exposure^2,3^. Clinically, sequelae of TBI can include PTSD, major depressive disorder (MDD) and anxiety disorders, but the efficacy of treatments for these complications is limited^4,5^. For example, first-line therapies for PTSD are less effective in veteran populations^6–8^ and overall remission rates of available treatments for these complications range from 20% to 40% (refs. ^9,10^). Perhaps most concerningly, veterans make up 20% of suicides in the United States of America despite making up only 6.4% of the general population^11^. Exposure to repeated blasts can result in changes to the brain, including to structure, functional connectivity, cerebral blood flow and white matter^12–14^. The sequelae of TBI may also include both subjective and objective changes in memory, attention, processing speed and executive functions that can substantially impact quality of life^13,15–18^. Desperate for relief, some veterans have begun seeking underexplored therapies that are not currently available in the United States, such as the oneirogenic alkaloid ibogaine, but data on the effectiveness and safety of this treatment are lacking.

Ibogaine is derived from the root bark of the *Tabernanthe iboga* shrub and related plants and is traditionally used in African religious, spiritual and healing ceremonies^19^. Therapeutic dosing leads to dreamlike states of consciousness that facilitate a longer period of self-reflection and evaluation. Pharmacologically, ibogaine and its principal metabolite noribogaine demonstrate moderate-to-weak affinity for a number of neurotransmitter receptors including *N*-methyl-d-aspartate, κ and μ opioid, σ-1 and σ−2, nicotinic acetylcholine, serotonin transporter and dopamine transporter, among others^20–22^. Ibogaine also increases the transcription of neurotrophic factors including brain-derived neurotrophic factor and glial cell line-derived neurotrophic factor^23^ and increases cortical neuron dendritic arbor complexity in vitro^24^. This unique pharmacology results in ibogaine’s classification as an atypical psychedelic^25^ and the aforementioned nature of the experience has led to it being termed an ‘oneirogen’^26–28^. Although both are appropriate, we use the latter term throughout to emphasize these distinguishing characteristics.

Importantly, ibogaine is classified by the Controlled Substances Act as a Schedule I substance, indicating that there is no currently accepted medical use and a high potential for abuse according to the US Drug Enforcement Agency. Such legal restrictions have limited research, as have concerns related to neuro- and cardiotoxicity^20,29^. With regard to the former, only transient ataxia has been reported in humans^20^. In the case of the latter, however, lengthening of the time of ventricular depolarization and repolarization (Q–T interval prolongation), with instances of subsequent fatal arrhythmia, has occurred^29^. High doses of ibogaine, pre-existing conditions, drug–drug interactions and lack of vital sign monitoring may have played critical roles in these cases^20^. Magnesium supplementation has been shown to reduce the Q–T interval^30^ and magnesium can protect against Q–T interval prolongation when coadministered with medications that ordinarily would have such an effect^31^, raising the possibility that its coadministration with ibogaine may offer cardioprotection and improved safety.

To date, ibogaine research has focused predominantly on its potential as a treatment for substance use disorders (SUDs)^32–36^. Some studies of ibogaine for SUDs have also noted improvements in self-reported measures of mood^37^, but no studies have prospectively validated effects on mood with more rigorous clinician-rated instruments. US SOVs have noted subjective improvements after ibogaine^33,38^. SOFs are deployed at a greater pace and to higher intensity combat than conventional military, exposing them to greater allostatic load and risk of injury, including from blast exposure^39,40^. This, in turn, has been proposed to result in a unique pattern of physical, cognitive, behavioral, psychiatric and endocrine-related problems that negatively impact ongoing functioning across several domains^40,41^. Although studies reporting specifically on SOV treatment outcomes are lacking^42^, individuals with combat-related TBI and comorbid conditions including PTSD and depression may have higher suicide risk^43,44^.

Given this substantial burden of ongoing disability and suicide risk in SOVs, additional treatment options are needed. In the present study, we present initial results from a prospective study examining the safety and efficacy of the Magnesium–Ibogaine: the Stanford Traumatic Injury to the CNS protocol (MISTIC) in SOVs with a history of predominantly mild TBI and repeated blast/combat exposures and subsequent development of functional limitations and psychiatric symptoms.

## Results

### Demographics

**Fig. 1:**
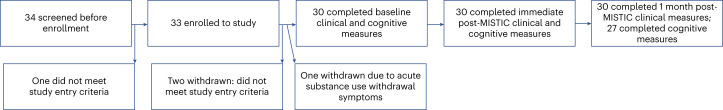
CONSORT diagram. Participant numbers at screening, enrollment and throughout progression of the study.

As detailed in the CONSORT (Consolidated Standards of Reporting Trials) diagram (Fig. 1), 34 SOVs were screened, 33 initially enrolled and ultimately 30 were eligible and completed baseline and posttreatment assessments between November 2021 and September 2022. All participants were male, reflecting the usual gender breakdown of SOFs. Fifteen participants met the criteria for MDD, 14 for an anxiety disorder and 23 for PTSD. Participants received 12.1 ± 1.2 (mean ± s.d.) mg kg^−1^ of oral ibogaine. Additional demographic information is provided in Table 1.

**Table 1:** Baseline demographics and sample characteristics

Baseline demographics and characteristics		Diagnosis according to MINI DSM-5	*n*
Total *n*	30	PTSD	23
Age	44.9 ± 7.5	PTSD with dissociative symptoms	6
		Major depressive disorder	15
IQ estimate (two-subtest estimate of full-scale intelligence quotient)	114 ± 10.3	Anxiety disorder^d^	14
Combat Exposure Scale^a^	29.6 ± 5.2	Alcohol use disorder	15
Number of TBIs^b^	38.6 ± 52.4	Other SUD^e^	6
Number of combat deployments	5.5 ± 3.0	Race and ethnicity	
Time since military discharge (years)	7.7 ± 4.8	White	26
TBI severity (mild, moderate, moderately severe)^c^	28, 1, 1	Biracial (white and Native American)	2
Number with past suicidal ideation	19	Native American	1
Number with past suicide attempt	7	Hispanic	1

All statistics are presented as mean ± s.d. MINI, Mini International Neuropsychiatric Interview; DSM-5, *Diagnostic and Statistical Manual of Mental Disorders*, 5th edn.

^a^A higher score on the CES means higher combat-related stress.

^b^Number of TBIs was assessed using the BAT-L.

^c^TBI severity was assessed using the OSU-TBI.

^d^Includes generalized anxiety disorder (8), panic disorder (6), agoraphobia (4) and social anxiety disorder (3) (4 participants had >1 anxiety disorder).

^e^Includes pain medication (1), stimulants (3) and cannabis (2).

### Primary outcome

**Fig. 2:**
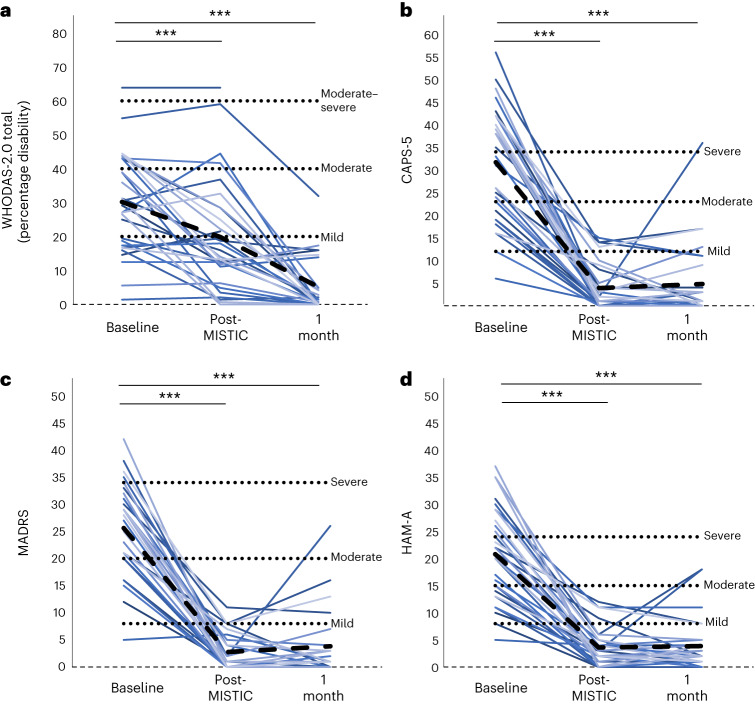
Primary, secondary and exploratory outcomes. **a**–**d**, Baseline and follow-up results in WHODAS-2.0 total (**a**), CAPS-5 (**b**), MADRS (**c**) and HAM-A (**d**). Individual colored lines represent individual participants. The dashed black line represents the mean. LME models were used for each comparison with FDR correction applied for determination of significance. ^***^P_FDR_ < 0.001.

The prespecified primary outcome was a change in the World Health Organization Disability Assessment Schedule 2.0 (WHODAS-2.0)^45^ from baseline to posttreatment. As illustrated in Fig. 2a and further detailed in Table 2, a linear mixed effect (LME) model revealed that the WHODAS total score decreased significantly (*P*_corrected_ < 0.001) from 30.2 ± 14.7 (mild-to-moderate disability) at baseline to 19.9 ± 16.3 (borderline no-to-mild disability) at the immediate posttreatment evaluation (Fig. 2a) with effect size (Cohen’s *d*) of 0.74. The improvement was statistically significant across all subscales (Extended Data Table 1), with the greatest effect size noted for the cognition domain (*P*_corrected_ < 0.001; *d* = 0.96).

**Table 2:** Baseline and follow-up statistics of WHODAS-2.0

Baseline and follow-up statistics													
	Baseline	Post-MISTIC	Baseline versus post-MISTIC					1 month		Baseline versus 1 month			
			*F*^a^		*P*_FDR_		*d*			*F*^a^	*P*_FDR_		*d*
WHODAS-2.0 total	30.2 ± 14.7	19.9 ± 16.3	20.38		<0.001		0.74	5.1 ± 8.1		85.85	<0.001		2.20
CAPS-5	31.7 ± 12.5	3.9 ± 4.8	206.14		<0.001		2.30	4.8 ± 7.9		191.77	<0.001		2.54
MADRS	25.6 ± 8.7	2.8 ± 3.3	249.72		<0.001		2.65	3.8 ± 6.0		229.28	<0.001		2.80
HAM-A	20.8 ± 8.5	3.6 ± 3.4	164.24		<0.001		2.06	3.9 ± 4.6		164.24	<0.001		2.13
	Percentage reporting SI	Percentage reporting SI	*X*^2^		*P*_FDR_		–	Percentage reporting SI		*X*^2^	*P*_FDR_		–
SI (MADRS Q10)	47	0	18.26		<0.001		–	7		12.27	<0.001		–

Percentage reduction, response and remission rates						
	Percentage reduction versus baseline		Response rate (%)		Remission rate (%)	
	Post-MISTIC	1 month	Post-MISTIC	1 month	Post-MISTIC	1 month
CAPS-5	88 ± 15	88 ± 17	97	100	86	86
MADRS	87 ± 23	87 ± 17	100	97	83	83
HAM-A	81 ± 19	81 ± 21	97	93	86	83

All results are presented as mean ± s.d. LME models were used for each comparison with FDR correction applied for determination of significance.

^a^Degrees of freedom (d.f.) were (1.72) for WHODAS-2.0 and (1.75) for CAPS-5, MADRS and HAM-A.

### Secondary outcomes

We also assessed change in WHODAS from baseline to 1 month after treatment. Again, as illustrated in Fig. 2a and further detailed in Table 2, the WHODAS total score decreased significantly to 5.1 ± 8.1 (no disability) (*P*_corrected_ < 0.001; *d* = 2.20).

Additional prespecified secondary outcomes included posttreatment changes on the Clinician-Administered PTSD Scale for DSM-5 (CAPS-5)^46^, the Montgomery–Åsberg Depression Rating Scale (MADRS)^47^ and the Hamilton Anxiety Rating Scale (HAM-A)^48^. LME models revealed statistically significant lowered CAPS-5, MADRS and HAM-A scores immediately post-MISTIC and at the 1-month follow-up (Fig. 2b–d and Table 2), with *d* > 2.0 in all cases.

### Safety

There were no unexpected or serious treatment-emergent side effects and there were no instances of bradycardia, tachycardia, clinically meaningful (that is, qualitatively detectable on monitoring) Q–T prolongation or hemodynamic instability. All participants experienced transient cerebellar signs such as mild ataxia and intention tremor that resolved within 24 h. While experiencing the oneirogenic effects of MISTIC, 12 participants (40%) were treated for headache, 7 (23%) for nausea, 3 (10%) for anxiety, 2 (7%) for hypertension and 1 (3%) for insomnia.

### Exploratory outcomes

To further assess changes in psychiatric symptoms identified by the models, we calculated the mean percentage reduction, response rate and remission rate according to the CAPS-5, MADRS and HAM-A (Table 2). Response on the CAPS-5, MADRS and HAM-A was defined as a reduction of at least 10 points^49^, 50%^50^ and 50%^51^, respectively; remission was defined as a loss of diagnosis and a total score <12 (ref. ^49^), total score <8 (ref. ^50^) and total score <8 (ref. ^51^), respectively. Of note, one participant’s baseline scores met criteria for remission on all three scales and so were excluded from the calculation of response and remission rates, leaving 29 participants in these specific analyses. As shown in Fig. 2 and Table 2, mean percentage reductions were at least 81%, response rates at least 93% and remission rates at least 83%. Effect sizes were all >2.0.

We also performed an exploratory analysis of the effect of MISTIC on suicidal ideation (SI). We compared the proportion of participants with a score ≥1 on the MADRS SI item and found a statistically significant reduction from 47% at baseline to 0% and 7% at posttreatment and 1-month follow-up, respectively (Table 2).

**Fig. 3:**
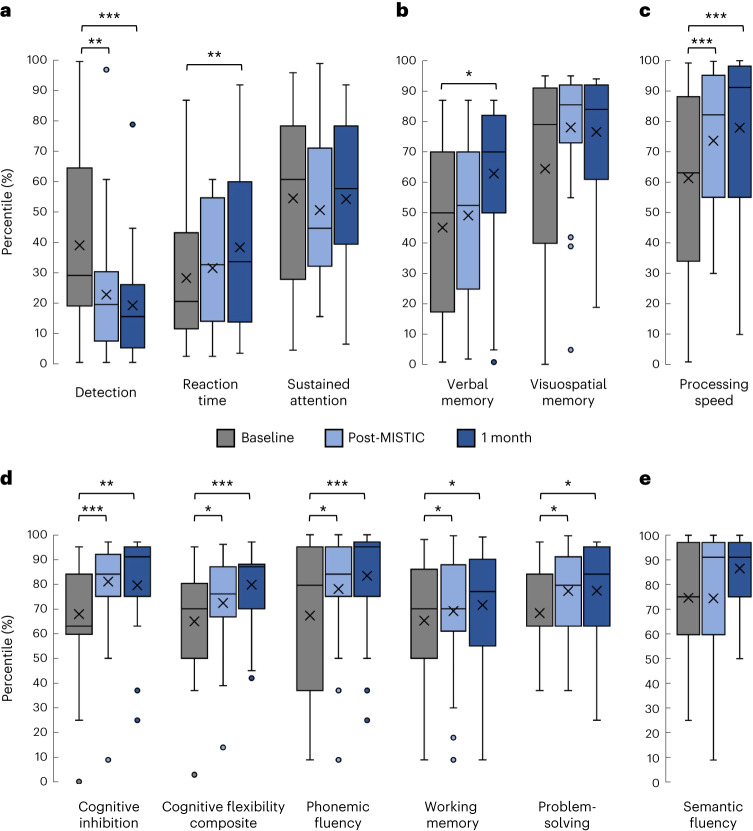
NPT. **a**–**e**, Baseline and follow-up results in percentile relative to age-matched peers in sustained attention (lower scores for detection represent improvement) (**a**), learning and memory (**b**), processing speed (**c**), executive function (**d**) and language (**e**). The *y* axis represents the percentile and the *x* axis the mean; the middle line represents the median, the whisker lines the interquartile range (IQR) and single dots participants with a score >±1.5 IQR. LME models were used for each comparison with FDR correction applied for determination of significance. ^*^P_FDR_ < 0.05; ^**^P_FDR_ < 0.01; ^***^P_FDR_ < 0.001. See Table 3 for *P* values and for the specific test item(s) included in each construct. The *n* for each construct at baseline, post-MISTIC and 1-month time points, respectively: detection, reaction time and sustained attention: 24, 28, and 20; verbal memory and working memory: 29, 30 and 27; visuospatial memory, processing speed, cognitive inhibition, cognitive flexibility composite, phonemic fluency and semantic fluency: 30, 30 and 27; problem-solving: 27, 30 and 27.

To assess for any cognitive effects of MISTIC, particularly given the history of TBI in study participants, a neuropsychological battery was administered to participants at all three time points (see Table 3, Fig. 3 and Extended Data Table 2 for pre–post score comparisons). The results indicated statistically significant improvements in processing speed with large effect sizes (*d* = 0.97–1.34) and executive functioning (including inhibition, cognitive flexibility, problem-solving, phonemic fluency and working memory, with effects ranging from small to large: *d* = 0.31–1.22), both immediately post-MISTIC and at the 1-month follow-up. Mean performances on these tests moved from the average to the high average score range relative to same-age peers and, in all but one instance, phonemic fluency was high average at baseline and improved to the superior range relative to same-age peers at the 1-month follow-up (*d* = 1.11). Learning and memory tests showed a significant improvement in visual memory at both time points and in verbal memory at the 1-month follow-up. Sustained attention showed a significant improvement in accuracy (detection) at both time points with large effect sizes (*d* = 0.86–1.05) and a weak but significant slowing of reaction time (*d* = 0.29–0.52), consistent with a prioritization of accuracy over speed and reduced impulsivity. No significant performance changes were observed in language (semantic fluency). No declines were noted across any performance domain.

**Table 3:** Baseline and follow-up statistics of NPT

NPT										
Neuropsychological construct	Neuropsychological test item	Baseline	Post-MISTIC	Baseline versus post-MISTIC			1 month	Baseline versus 1 month		
				*F*^a^	*P*_FDR_	*d*		*F*^a^	*P*_FDR_	*d*
Sustained attention										
Detection^b^	CPT-3 detection	46.6 ± 10.3	41.2 ± 8.2	13.42	0.002^*^	1.05	39.5 ± 7.5	19.40	<0.001^*^	0.86
Reaction time	CPT-3 reaction time	43.0 ± 7.7	44.1 ± 6.8	1.17	0.330	0.29	46.4 ± 8.1	9.42	0.008^*^	0.52
Sustained attention	CPT-3 hit reaction time block change	51.5 ± 8.8	50.8 ± 7.9	0.02	0.888	0.02	51.2 ± 7.7	0.48	0.550	0.29
Learning and memory										
Verbal memory	HVLT-R	47.4 ± 10.1	49.0 ± 9.2	0.34	0.595	0.17	53.1 ± 8.8	6.32	0.026^*^	0.47
Visuospatial memory	BVMT-R	53.9 ± 11.4	58.8 ± 7.1	9.33	0.008	0.50	58.3 ± 6.6	4.28	0.056	0.32
Processing speed										
Processing speed	PSI (WAIS-IV)	53.8 ± 10.6	59.2 ± 9.7	27.65	<0.001^*^	0.97	61.6 ± 10.7	43.51	<0.001^*^	1.34
Executive function										
Cognitive inhibition	D-KEFS color/word interference, condition 3	55.1 ± 8.8	59.9 ± 6.4	21.33	<0.001^*^	1.22	59.9 ± 7.5	15.68	0.001^*^	0.62
Cognitive flexibility composite	Mean of: (1) D-KEFS TMT4; (2) D-KEFS color/word interference, condition 4; (3) D-KEFS verbal fluency, category switching	54.0 ± 8.0	56.6 ± 5.7	4.72	0.046^*^	0.43	59.3 ± 5.0	17.61	<0.001^*^	0.74
Phonemic fluency	D-KEFS verbal fluency	57.0 ± 11.7	60.8 ± 10.3	7.53	0.016^*^	0.52	64.0 ± 10.1	21.79	<0.001^*^	1.11
Working memory	WMI (WAIS-IV)	55.1 ± 8.3	57.0 ± 9.5	5.20	0.037^*^	0.37	57.6 ± 9.2	5.63	0.033^*^	0.31
Problem-solving	D-KEFS TT, total achievement score	55.7 ± 6.4	59.1 ± 7.1	5.44	0.034^*^	0.49	59.5 ± 7.9	6.29	0.026^*^	0.44
Language										
Semantic fluency	D-KEFS verbal fluency	60.4 ± 11.4	60.2 ± 12.2	0.18	0.690	0.02	63.6 ± 7.8	1.97	0.205	0.24

All results are presented as mean ± s.d. Neuropsychological testing (NPT) scores are represented as a T score (mean of 50, s.d. of 10). Unless stated otherwise, a higher score represents better performance. LME models were used for each comparison with FDR correction applied for determination of significance. BVMT-R, Brief Visuospatial Memory Test—Revised; HVLT-R, HVLT—Revised.

^a^Degrees of freedom were: (1.60) for CPT-3; (1.72) for working memory, verbal memory and problem-solving; and (1.73) for visuospatial memory, cognitive inhibition, cognitive flexibility, phonemic fluency, semantic fluency and processing speed.

^b^Lower score indicates better performance.

*Results were considered statistically significant only if the FDR-corrected P values both of the main effect (as reported in Extended Data Table 2) and the specific contrast (baseline versus post-MISTIC or baseline versus 1 month, respectively) were <0.05.

### Sensitivity analyses

To ensure that individuals without the relevant comorbidity were not driving our findings of reductions in PTSD, depression and anxiety symptoms, we also repeated our calculations in subgroups that excluded all participants who, at baseline, did not meet the criteria on the structured diagnostic interview for the disorder assessed by the scale (for example, PTSD for CAPS-5). The results were similar, with remission rates at 1-month follow-up of at least 67% (Extended Data Table 3). Analogously, we repeated our assessment of the effect of MISTIC on SI, including only participants with non-zero SI at baseline on the MADRS. Results again were largely unchanged (Extended Data Table 3).

Finally, to determine whether the participants with more severe TBI history may be biasing results, we also performed a sensitivity analysis excluding the participants with non-mild TBI; results again were largely unchanged (Extended Data Table 4), with remission rates at 1-month follow-up of at least 85%.

## Discussion

In summary, we prospectively investigated the safety and efficacy of MISTIC for SOVs with a history of TBI and repeated blast/combat exposures. At baseline, study participants experienced clinically meaningful levels of disability, PTSD, depression and anxiety. After MISTIC, participants showed a remarkable reduction in these symptoms with large effect sizes (Cohen’s *d* > 2 on clinician-rated psychiatric assessments) and the benefits were sustained at the 1-month follow-up. Indeed, disability measures continued to improve and psychiatric symptom remission and response rates 1 month post-MISTIC remained high. Neuropsychological testing (NPT) revealed areas of improvement after treatment, particularly in processing speed and executive function, without any detrimental changes observed. With regard to safety, no serious or unexpected adverse events (AEs) occurred and management of AEs was uncomplicated.

This is possibly the first study to report evidence for a single treatment with a drug that can improve chronic disability related to repeated TBI from combat/blast exposures. Moreover, there is no currently available US Food and Drug Administration (FDA)-approved treatment for chronic sequelae of combat-related TBI. Current treatment options include cognitive rehabilitation, psychotherapy and medications that target specific symptoms, but there is limited evidence of efficacy^52–54^. Given the alarming rates of suicide in veterans^11^, as well as evidence that military-related TBI increases the risk of suicide in veterans^55^ (as TBI also does in the general population^56^), the substantial reduction in SI that we observed—which must be interpreted cautiously as an exploratory analysis—is noteworthy. TBI also is associated with increased impulsivity^53^, a well-known risk factor for suicide^57^, and MISTIC resulted in a measurable improvement in cognitive inhibition.

Although outside the context of TBI and veterans, our findings are consistent with previous studies suggesting benefits of treatment with psychedelic substances across several psychiatric disorders^19,33,36^. Recent studies of 3,4-methylenedioxymethamphetamine (MDMA)-facilitated psychotherapy, for example, showed promise in the treatment of PTSD^49,58,59^. Similarly, psilocybin has demonstrated improvements in depression, substance use and anxiety^60–63^. Other substances such as lysergic acid diethylamide (LSD) and ayahuasca have also shown notable improvements in depression and anxiety for most patients^64–66^.

Importantly, the present study was not a randomized controlled trial (RCT) and participants elected to travel internationally for the treatment. As such, we cannot exclude the possibility that the therapeutic benefits were a result of expectancy rather than MISTIC. Similarly, the complementary therapeutic approaches available to SOVs during their stay in Mexico may have played a role in the therapeutic benefit that we observed, because other similar approaches with veterans^67,68^ have demonstrated benefits, albeit considerably smaller than those that we found.

Although future placebo-controlled RCTs may help to establish the potential therapeutic benefits of ibogaine and the MISTIC protocol, the interpretation of placebo-controlled RCTs of psychedelic medicines is limited by the fact that very few studies^65,69^ have suggested that their blinds may have been intact. In the case of ibogaine, its unique oneirogenic effects and the relatively long duration of the experience (see Methods for further details) imply that attempts to perform a blinded RCT will experience similar challenges.

We attempted to further assess the contribution of placebo effects to our results by analyzing NPT. NPT is relatively insensitive to such effects^70^, with documented placebo effects on subjective performance, but not objective scores^71^. Furthermore, even when placebo effects have been reported on cognitive task performance, generally weak and short-term effects have been noted. For example, Parong and colleagues^72^ found that providing positive compared with negative expectations led to significant but weak effects of cognitive training on working memory, task switching and nonverbal reasoning (and not on other cognitive domains that they tested). These effects did not survive a short delay, however, suggesting that any placebo effects are short-lived. In our study, NPT revealed either improvement or no change, with the former most notable for processing speed, phonemic fluency and attentional accuracy. Thus, although the present study was not controlled, it is unlikely that the observed large, persistent improvements on NPT are due to placebo alone. In addition, the lack of any observed worsening is reassuring from a safety perspective, particularly given previous concerns about cerebellar toxicity with ibogaine^20,73^. We found no evidence of decline in psychomotor skills, language, executive functions or visuospatial abilities, all of which have been associated with cerebellar function^74,75^.

One limitation of NPT is its potential sensitivity to practice effects. In the present study, we attempted to minimize this by following data-driven recommendations^76^, including utilizing alternative forms of tests whenever available and favoring tests with low-to-no practice effects. In their meta-analysis of practice effects in NPT, Calamia et al.^70^ recommended considering practice effects per test, and not per domain, owing to test-specific factors. Although practice effects are expected for the Hopkins Verbal Learning Test (HVLT) and Delis–Kaplan Executive Function System (D-KEFS) Tower Test (TT) and verbal fluency used in the present study, for instance, only weak practice effects are expected for D-KEFS measures of inhibition, switching, Wechsler Adult Intelligence Score (WAIS) working memory and processing speed subtests^70^ and the Conners Continuous Peformance Test 3 (CPT-3) of sustained attention^77^. The benefits that we observed on these tests are, then, unlikely to result from practice effects.

Although the current results are promising, additional research is needed to address some clear limitations. Most importantly, and as discussed in detail above, the study was not controlled and so the relative contribution of any therapeutic benefits from non-ibogaine elements of the experience, such as complementary treatments, group activities, coaching, international travel, expectancy or other nonspecific effects, cannot be determined. Also, TBI and resulting functional disability were only mild in severity, on average, although PTSD, depression and anxiety symptom mean severities were in the moderate range at baseline. Although improvements were sustained for most participants at 1 month, long-term data are necessary to determine the durability of the effects, particularly as several participants experienced recurrence of notable psychiatric symptoms between the immediate post and 1-month time points; in the cases of at least two individuals, substantial psychosocial stressors were encountered on their return home that may have played a role in the decrease in durability. Importantly, no participants experienced any worsening of PTSD, depression or anxiety compared with baseline, and even the participants with the most prominent relapses still experienced >30% symptom improvement at the 1-month mark compared with baseline. In addition, our sample size was modest, although we note that it compares favorably with a number of other pilot studies of relevance^78–84^. We also believed that it was necessary to balance our desire for a larger sample with the importance of providing prompt preliminary safety and efficacy data to other SOVs who are considering this treatment given their potentially vulnerable status. Another limitation of our study is that the current sample was highly homogeneous, consisting mostly of white men from elite military units who tended to be in above-average physical condition. Although the demographics included here are reasonably representative of SOVs^85,86^, a study examining the safety and efficacy of MISTIC in a more diverse and medically complex population would be required to assess the generalizability of our findings beyond SOVs. Last, although our exploratory analysis suggested a beneficial effect of MISTIC on SI, further investigation with scales specifically designed to measure suicidality are required before any conclusions may be drawn.

In summary, our study provides initial evidence to suggest that MISTIC could be a powerful therapeutic for the transdiagnostic psychiatric symptoms that can emerge after TBI and repeated exposure to blasts and combat, including suicidality, but replication of our findings is needed, particularly in non-mild TBI cases. Considering that the average time since discharge from the military in our sample was nearly 8 years, these findings further suggest that MISTIC may be effective even when administered years after the injuries. Our results also raise the possibility that this therapy may be beneficial in other populations suffering from sequelae of repeated head trauma^87,88^. Importantly, our results indicate that ibogaine can be administered safely to an SOV population when combined both with magnesium and with appropriate screening, precautions and medical monitoring. Last, concerns that the use of certain psychedelics as therapeutics risks fostering a new addiction^89^ are mitigated by ibogaine’s apparent anti-addictive properties^32^. Although these conclusions must be considered preliminary, they support the need for further testing of MISTIC in larger, controlled trials.

## Methods

### Inclusion and ethics

All research procedures were approved by the Stanford University Institutional Review Board (IRB). We complied with all relevant ethical regulations. Written informed consent was obtained from all participants as further described below. Roles and responsibilities were agreed on among authors and collaborators. The trial was preregistered at ClinicalTrials.gov (NCT04313712) and osf.io (https://osf.io/24trc/).

### Participants

Study participants were 30 male SOVs who had independently scheduled themselves for MISTIC at Ambio Life Sciences in Mexico—where ibogaine use is not restricted—after being approved for a grant by a nonprofit organization, Veterans Exploring Treatment Solutions (VETS), Inc. Stanford played no role in ibogaine administration, as further noted below in the details about the consent process and, accordingly, no investigational new drug application with the US FDA was required by the Stanford University IRB. Ambio conducts its own application process and medical screening, including routine blood work, electrocardiogram (ECG) and instruction to discontinue certain medications with potentially concerning drug–drug interactions. These include diuretics, CYP2D6-inhibiting medications, serotoninergic medications (that is, any that may increase risk of serotonin syndrome), calcium channel blockers, β-blockers, benzodiazepines, stimulants, corticosteroids and all psychiatric medications. Once scheduled, participants were informed of the present study and, if interested, referred to the Stanford study team.

Potential participants were then screened by the Stanford study team for eligibility. Participants were eligible if they were veterans aged between 18 and 70 years, were able to provide informed consent, had a history of head trauma, combat or blast exposure, had no contraindication to magnetic resonance imaging (MRI) and were able to travel to Stanford for relevant study time points (travel and accommodation were funded by VETS, Inc.). Exclusion criteria included a history of a neurological disorder (excluding sequelae of TBI), a history of any psychotic symptoms or disorders, being at risk for suicidal behavior during the study in the judgment of the investigator, having a clinical abnormality on screening physical exam that could affect safety or study integrity, recent or concurrent participation in another study with a drug or device, a history of cardiovascular, liver or kidney problems, pregnancy or any other condition that would affect the individual’s ability to safely participate.

Racial/ethnic identity was determined by the participants using classification terms provided by the researchers. Classification terms were: American Indian or Alaska Native; Asian; Black or African American; Native Hawaiian or Other Pacific Islander; white; Hispanic or Latino (ethnicity); not Hispanic or Latino (ethnicity).

Gender was determined by the participants using classification terms provided by the researchers. Classification terms were: ‘male’, ‘female’ or ‘other’.

All participants signed the informed consent form on enrollment. The consent process was video recorded and included asking participants to clarify their understanding of the study, their understanding of their role, their rights as study participants, their expectations and whether or not their participation was coerced. A trained neuropsychologist performed the entire consenting process.

Highlights of the informed consent form that was reviewed and signed include:

- Participants: “Participants in this study are US citizens who have been referred by VETS, Inc. and who have had previous head trauma, blast or combat exposure and have independently and voluntarily opted to receive ibogaine exposure in Mexico.ˮ
- Purpose of research: “Previous research has reported some evidence that this compound can be used as a protective agent to help reduce or prevent brain damage. We would like to learn more about this compound to improve our understanding of the risks associated with its use. Exposure to this psychoactive compound can be unsafe, especially for individuals who have pre-existing heart conditions. Use of this compound is forbidden by the Food and Drug Administration (FDA).ˮ
- Voluntary participation: “Participation in this study is entirely voluntary.ˮ
- Duration of study involvement/procedures: all study visits with Stanford are described.
- Possible risks: “We are not supporting, facilitating or condoning use of ibogaine HCL. We are not providing any medical screening or supervision for the treatment that you have elected to undertake. Measurements we are taking are for research purposes but not for medical monitoring.ˮ
- Potential benefits: “There is no direct benefit to you for participating in the study. We cannot and do not guarantee or promise that you will receive any benefits from this study.ˮ

### Procedure

After enrollment, participants undertook initial baseline evaluations over a secure video platform with a clinical neuropsychologist between 2 months and 1 week before in-person assessments, including review of medical and psychiatric history, history of combat exposures, history of TBI and blast exposure, and a psychodiagnostic interview. All participants presented with a history of TBI, according to the Ohio State University Screening for TBI exposure (OSU-TBI)^90^ and the Department of Defense TBI classification^91^. In addition, to quantify blast exposure, the Boston Assessment of TBI—Lifetime (BTA-L)^91^ was administered.

Similar to other studies evaluating treatments that induce altered states of consciousness^49^, participants were also paired by VETS, Inc., with a licensed therapist familiar with and experienced in coaching patients undergoing ibogaine treatment for individual sessions that are structured in nature. Pretreatment coaching practices include intention setting, tools for managing expectations and reducing anxieties associated with treatment. After treatment, coaches assist with processing emotions, helping to define meaning and integrating insights from the treatment experience into participants’ everyday lives. Coaching does not involve diagnosing, delving into past traumas or medication-based approaches to healing.

Then, 2–3 d before scheduled treatment, small groups of two to four participants traveled to Stanford University, where they underwent in-person evaluation that included self-report measures and clinical and neuropsychological assessment by a trained assessor. They then traveled independently to the treatment site in Mexico for MISTIC as described below. Additional therapeutic wellness activities available on site to complement the treatment included sweat lodge, massage, yoga, reiki, breathwork and meditation. Participants returned to Stanford for repeat evaluation 4–5 d after treatment and again 1 month later.

### MISTIC treatment at Ambio Life Sciences

The Ambio Treatment Center is located in the suburban Tijuana area. The center includes shared and private bedrooms, dining facilities and other communal areas. The treatment space is a large room containing mats spread out across the floor, where patients recline while under the effects of ibogaine. The room also contains the medical monitoring equipment and an adjacent nursing station contains all supplies and medications that may be needed for management. On arrival, participants were assessed by the clinic’s medical staff including blood work, ECG and urinalysis. A maximum of five patients were treated at the Ambio clinic at one time. Group preparatory and ceremonial activities took place. Day 2 involved additional group preparatory activities and an 8-h fast before the treatment, which began on the evening of day 2 and continued through day 3. Of note, no psychotherapy occurred during treatment, but support was offered by monitoring personnel if needed. Otherwise, the treatment experience was completely self-guided and patients were spatially separated from each other and wearing eye shades. Integration activities occurred on day 4 and participants returned to the United States on the evening of day 5 to return to Stanford University for the next study visit.

Ibogaine hydrochloride (98+% pure) used for treatment was synthesized in South Africa by Cape Analytical Service Laboratories from voacangine which was ethically sourced from *Voacanga africana* trees. With subjects in the fasting state, as noted above, the Ambio clinic personnel administered an intravenous infusion of 1 g of magnesium sulfate and an oral gastrointestinal protective agent 1–2 h before treatment. The oral ibogaine dosing protocol consisted of an initial test dose of 2–3 mg kg^−1^ of ibogaine. Depending on response, after ~40 min additional doses of ibogaine, up to a total of <14 mg kg^−1^, were administered within a total 2-h period. Approximately 12 h after administration of ibogaine, participants were administered an additional intravenous dose of magnesium sulfate, oral and intravenous antioxidants and metabolic supporting agents. Medical staff (MD, registered nurse or emergency medical technician) with advanced cardiovascular life support certification and extensive experience in administering ibogaine and monitoring treatment with it were onsite at a ratio of at least one member of staff to two patients throughout treatment for monitoring and management, but no specific coaching or psychological support was provided during treatment. For 12–16 h after ibogaine administration, blood pressure and pulse oximetry were monitored three times a day and the QTc was monitored visually via continuous 5-lead ECG. In one participant’s case, 4 mg kg^−1^ of booster dose was provided 12 h after the initial dose, given insufficient treatment intensity/duration as judged by clinic personnel; medical monitoring was extended accordingly.

### Treatment experience

Alper^92^ describes therapeutic dosing of ibogaine typically leading to three sequential stages beginning approximately 1–3 h after ingestion: ‘acute’ (~4–8 h), ‘evaluative’ (~8–20 h) and ‘residual’ (~24–72 h). Dreamlike states of consciousness begin during the acute stage, usually with closed eyes. Participants were able to visually orient themselves in the room as needed during their experiences. This acute stage leads into contemplation of the experiences from the previous stage. The residual stage involves reintegration with the environment as any lingering effects resolve.

### Structured assessments

#### MINI

The Mini International Neurodiagnostic Interview (MINI) is a structured diagnostic interview based on DSM-5^93^. It typically permits an experienced clinician to conduct a valid diagnostic interview with good inter-rater and test–retest reliability^94^.

#### SCID overview

The Structured Clinical Interview for DSM Disorders (SCID) overview is a semistructured review of an individual’s history with respect to health, mental health, occupation/education, substance use and psychosocial setting^95^.

#### Combat exposure

The Combat Exposure Scale (CES) is a retrospective seven-item scale to quantify stress associated with level of combat exposure. Each item has five response levels. Total scores can be interpreted as light (0–8), light–moderate (9–16), moderate (17–24), moderate–heavy (25–32) or heavy (33–41)^96^.

#### BAT-L

The BAT-L is a semistructured interview to quantify the incidence and severity of TBI in one’s lifetime. This instrument is validated for use with veterans^91^.

#### OSU-TBI

The OSU-TBI—Short Form is a structured interview to review an individual’s incidence of TBI in their lifetime. The Short Form version takes approximately 5 min to administer. The original form is reliable and validated in populations at risk for TBI. The short form carries over well-validated indices from the previous version^90^.

#### Function

The WHODAS-2.0 assesses the impact of health conditions across six life domains (cognition, mobility, self-care, interpersonal, life activities and community participation) and is sensitive to change over time^97^. Each item is rated on a scale ranging from no problems to extreme problems^45^ in the past 30 d. To capture interindividual variability in disability, we used the WHODAS complex scoring method. Raw scores are converted to a metric ranging from 0 (no disability) to 100 (full disability), by calculating the ratio of the participant’s score relative to the maximum possible score in each domain as well as to the total score^45^. A score of 20–39% is considered mild, 40–59% moderate, 60–79% moderate–severe and 80–100% severe.

#### PTSD symptoms

The CAPS-5 is considered the gold standard in evaluating the intensity and frequency of PTSD symptoms across the diagnostic criteria of intrusions, avoidance, negative cognitions or mood and arousal, as well as the presence and severity of dissociative specifiers (depersonalization and derealization). The past-week version is a 30-item structured interview of PTSD symptoms over the past week using a 0 (‘Absent’) to 4 (‘Extreme/Incapacitating’) scale, with possible total scores ranging from 0 to 80. The score range 23–34 is considered to be moderate PTSD, whereas a higher score represents severe PTSD^46^. Response on the CAPS-5 was defined as a reduction of at least 10 points^49^. Remission was defined as loss of diagnosis and a total score <12 (ref. ^49^).

#### Depression symptoms

The MADRS is a clinician-administered, ten-item scale assessing the severity of depression symptoms in the past week. Items are rated on a scale of 0 (no abnormality) to 6 (severe)^47^. A total score of 0–6 indicates no depression, 7–19 mild depression, 20–34 moderate depression, 35–59 severe depression and 60+ very severe depressive symptoms^98^. Response on the MADRS was defined as a reduction of total score by at least 50% of baseline. Remission was defined as a total score <8 (ref. ^50^).

#### Anxiety symptoms

The HAM-A includes 14 items assessing both psychic and physical symptoms of anxiety in the past week. Items are rated on a scale from 0 (no symptoms) to 4 (severe symptoms)^99^. Matza et al.^100^ identified optimal total score ranges to represent no or minimal anxiety (≤7), mild (8–14), moderate (15–23) and severe anxiety symptoms (≥24). Response on the HAM-A was defined as a reduction of total score by at least 50% of baseline. Remission was defined as a total score <8 (ref. ^51^).

### Neuropsychological battery

The neuropsychological test battery was administered by or under the supervision of a neuropsychologist. Tests and time points of administration are outlined below. Alternative forms were used when available at different time points, as noted below.

#### WASI-II two-subtest estimate of full-scale intelligence quotient

The Wechsler Abbreviated Scale of Intelligence, 2nd edtion^101^ two-subtest version was administered at baseline only to provide an estimate of baseline intellectual functioning (suitable for ages 6–90 years). Two subtests were administered:

1. Vocabulary: 31 questions requiring provision of definitions for words presented both visually and orally. Knowledge of vocabulary provides a representation of crystallized intelligence, understood to be more resistant to effects of neurological damage.
2. Matrix reasoning: 30 items providing a measure of visuospatial reasoning and pattern recognition.

#### WAIS-IV

The WAIS, 4th edn (WAIS-IV)^102^ is the gold standard for quantifying intellectual functioning. Four indices provide measures of different aspects of intellectual functioning (suitable for ages 16–90). Four subtests of the WAIS-IV were administered at baseline, immediately post-MISTIC and 1 month post-MISTIC, providing measures of two indices—the Working Memory Index (WMI) and the Processing Speed Index (PSI). Both working memory (ability to hold on to and mentally manipulate/update information) and information processing speed (ability to quickly and accurately process information) are measures of cognitive function and efficiency and may be susceptible to neurological damage.

- WMI—Digit Span Arithmetic subtests
  - Digit Span: increasingly long strings of numbers must be repeated forward, in reverse order and in sequential order. This test requires auditory attention as well as working memory.
  - Arithmetic: mental arithmetic problems of increasing challenge are presented verbally, which must be solved within a specified timeframe without writing information down.
- PSI—Symbol Search and Coding subtests
  - Symbol Search: the examinee must complete a visual discrimination task as quickly and as accurately as possible within a specified timeframe.
  - Coding: the examinee must write a symbol that is matched to a number for numbers that are presented alone, without its symbol, as quickly and as accurately as possible within a specified timeframe.

#### Hopkins Verbal Learning Test—Revised

The HVLT-R^103^ test is suitable for those between the ages of 16 years and 80+ years. The examinee must learn a list of words over three learning trials and then recall the list after a 20-min delay. The test provides measures of immediate recall, learning, delayed recall and recognition. There are six alternative forms of the test and each participant was administered an alternative form at the three different time points. Psychometrically, forms were clustered into groups of 1, 2 and 4, or 3, 5 and 6 (ref. ^103^). This test was administered at baseline, immediately post-MISTIC and 1 month post-MISTIC.

#### Brief Visuospatial Memory Test—Revised

The BVMT-R^104^ test is suitable for those aged between 16 years and 79 years. Examinees must learn an array of simple, geometric shapes over three learning trials and then recall the shapes after a 25-min delay. The test provides measures of immediate recall, learning, delayed recall and recognition. There are six alternative forms of the test. This test was administered at baseline, immediately post-MISTIC and 1 month post-MISTIC.

#### Delis Kaplan Executive Function System

The D-KEFS^105^ is the gold standard for testing executive functions in individuals aged 8–89 years. Four subtests were administered at baseline, immediately post-MISTIC and 1 month post-MISTIC.

- TMT: this test has five timed conditions: (1) visual scanning; (2) connecting numbers in order; (3) connecting letters in order; (4) alternating between connecting numbers and letters in order; and (5) psychomotor speed. Conditions 1, 2, 3 and 5 allow the examiner to identify whether a low score on condition 4 is related to one of the component skills in the other conditions. Condition 4 provides a measure of cognitive switching.
- Verbal fluency: (1) letter (phonemic) fluency: the examinee is asked to say as many words as possible that start with a given letter, within a specified timeframe. (2) Category (semantic) fluency: the examinee is asked to say as many words as possible from a given category within a specified timeframe. (3) Category switching: the examinee is asked to say as many words as possible, alternating between two given categories, within a specified timeframe. Condition 3 provides a measure of cognitive switching. There is one alternative form of the test and versions were alternated at different time points.
- Color/word interference: the examinee must, as quickly and accurately as possible: (1) name color patches; (2) read words denoting color names; (3) name the color of ink in which words denoting different colors are printed; and (4) respond according to specified rules that require the examinee to either read the word or name the dissonant ink color. Condition 3 provides a measure of cognitive inhibition and condition 4 provides a measure of both cognitive inhibition and cognitive switching.
- TT: the examinee must adhere to rules to build pictured towers using up to five disks of different sizes across three pegs, as efficiently as possible. This test provides measures of planning/organization and problem-solving efficiency.

#### Conners’ Continuous Performance Test Third Edition

The CPT-3^106^ is a computerized test, suitable for those aged 8 years and upward, that provides measures of inattention, impulsivity, sustained attention and vigilance. It was administered at baseline, immediately post-MISTIC and 1 month post-MISTIC. A letter is briefly presented on the computer screen at varying time intervals and the examinee must respond as quickly and accurately to one target letter only, among all other letters. There is a practice session before the test.

### Statistical analyses

As an observational study, no power calculation was performed. To assess the significance of post-treatment changes, LME models were used for each outcome measure. The false discovery rate (FDR)^107^ was applied to correct for multiple comparisons. All statistical analyses were performed in MATLAB R2021a. Figures were created using Excel 365. LME models were used for each outcome measure (WHODAS, CAPS-5, MADRS and HAM-A). Specifically, outcome measure scores served as the dependent variable and time point (baseline, post-MISTIC, 1-month follow-up) as the independent variable, with a fixed slope and random intercept; age, combat exposure (measured by the CES) and total number of TBIs were included in the model as fixed effects. The main effects of time point for each LME model are reported in Table 2 and Extended Data Tables 1 and 5. *F* and *P* values for contrasting post-MISTIC to baseline and 1-month follow-up were obtained using MATLAB’s hypothesis test on fixed-effect coefficients of LME models. LME models were also used to assess changes in neuropsychological function and separate FDR corrections were applied to these *P* values (Table 3 and Extended Data Table 2) and to the sensitivity analyses (Extended Data Tables 3–5). For the neuropsychological battery, scores used in the LME models were first converted to a common scale (T score: mean of 50 and s.d. of 10) for ease of comparison. All reported *P* values are two tailed. Comparison to baseline was marked statistically significant if the main effect and the contrast to baseline (post-MISTIC and 1-month follow-up) were significant at a level of *P*_FDR_ < 0.05.

Four participants did not complete the WHODAS-2.0 at the 1-month follow-up. At baseline, one participant did not complete items for working memory and verbal memory, three participants did not complete items for problem-solving and six participants did not complete the CPT-3. Post-MISTIC, two participants did not complete the CPT-3. At the 1-month follow-up three participants did not complete NPT and seven additional participants did not complete the CPT-3.

### Reporting summary

Further information on research design is available in the Nature Portfolio Reporting Summary linked to this article.

## Online content

Any methods, additional references, Nature Portfolio reporting summaries, source data, extended data, supplementary information, acknowledgements, peer review information; details of author contributions and competing interests; and statements of data and code availability are available at 10.1038/s41591-023-02705-w.

### Supplementary information

Reporting Summary

## Figures and Tables

**Extended Data Table 1:**
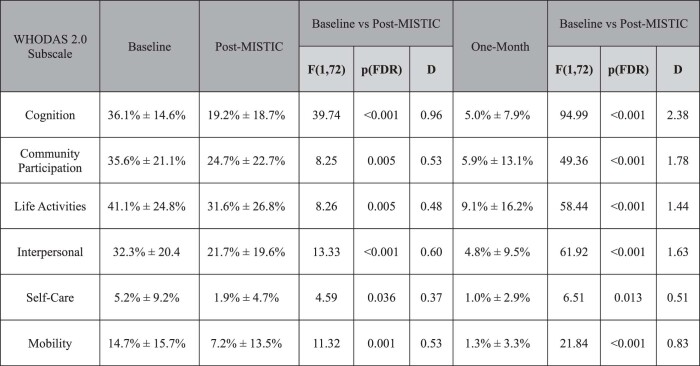
WHODAS-2.0 subscales

All results are presented as mean ± s.d. LME models were used for each comparison with FDR correction applied for determination of significance. Baseline *n* = 30 for all items.

**Extended Data Table 2:**
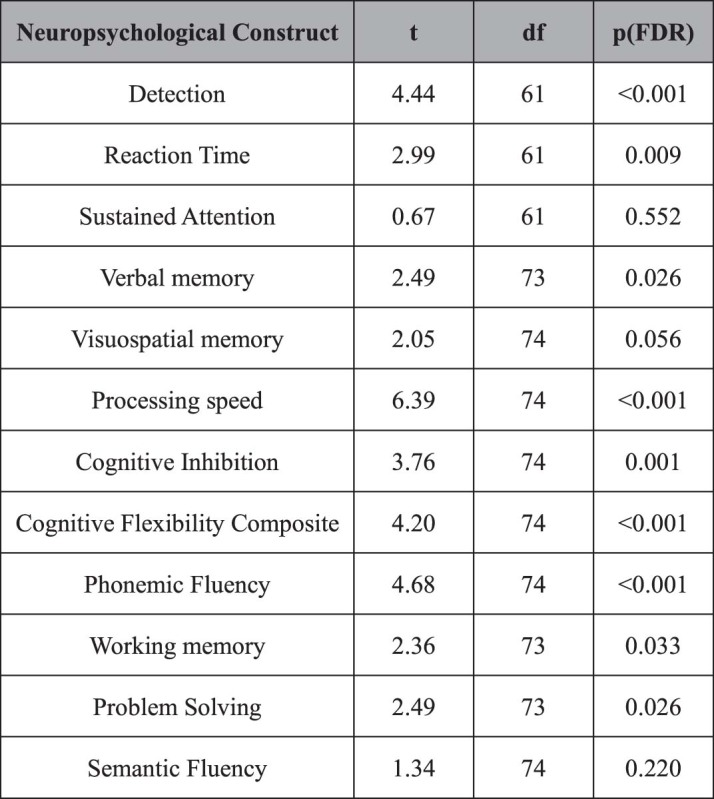
Statistical results (NPT)

Statistical results (*t*-test, d.f. and FDR-corrected *P* values)associated with main effects of time point for each LME model. LME models were used for each comparison with FDR correction applied for determination of significance. See the legend to Fig.3 for nos.

**Extended Data Table 3:**
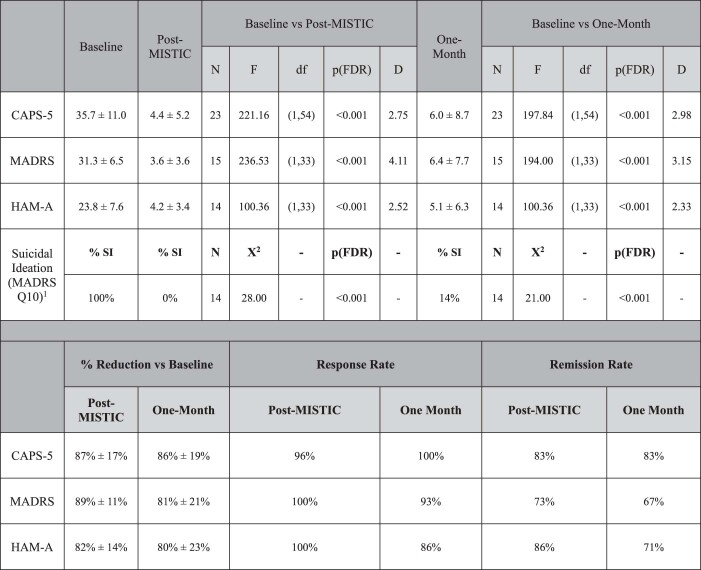
Sensitivity analyses including only participants meeting relevant diagnostic criteria

Sensitivity analyses including only participants meeting relevant diagnostic criteria (that is, PTSD for CAPS-5; MDD for MADRS; anxiety spectrum disorder (generalized anxiety disorder, panic disorder, social anxiety disorder and agoraphobia) for HAM-A) according to the MINI at baseline. All results presented as mean ± s.d. LME models were used for each comparison with FDR correction applied for determination of significance. Baseline *n* = 23 for CAPS, 15 for MADRS and 14 for HAM-A.

^1^For SI, analysis included only participants with measurable SI at baseline.

**Extended Data Table 4:**
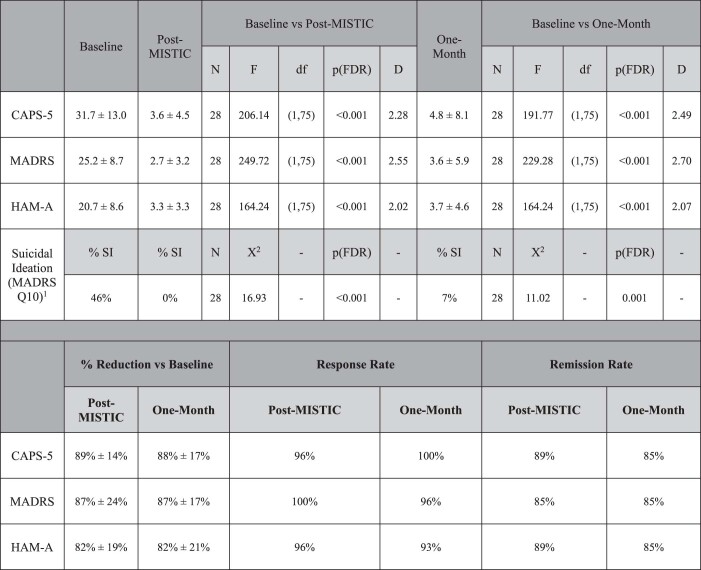
Sensitivity analyses including only participants with mild TBI

All results are presented as mean ± s.d. LME models were used for each comparison with FDR correction applied for determination of significance. Baseline *n* = 28 for all items. ^1^For SI, analysis included only participants with measurable SI at baseline.

**Extended Data Table 5:**
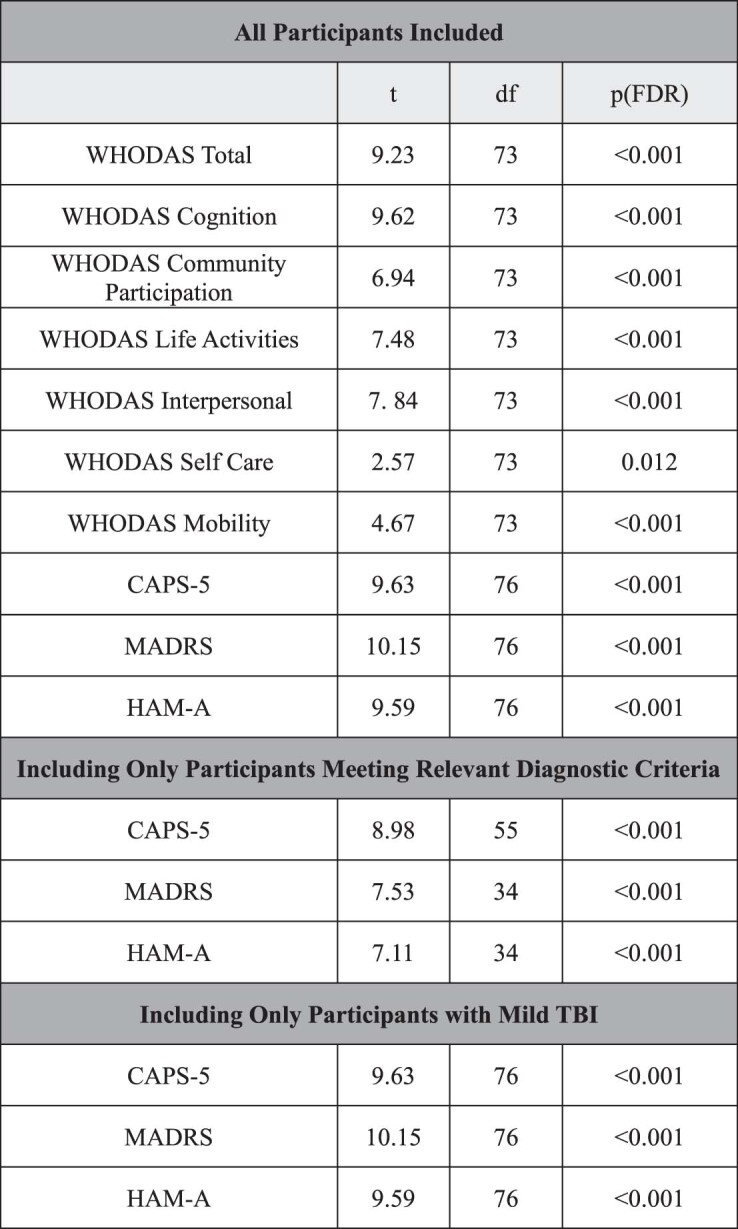
Statistical results (WHODAS and Clinical Scales)

Statistical results (*t*-test, d.f. and FDR-corrected *P* values) associated with main effects of time point for each LME model. Baseline *n* was 30 for all items including all participants (*n* = 23, 15 and 14 for CAPS-5, MADRS and HAM-A, respectively, for items including only participants meeting relevant diagnostic category; *n* = 28 for all items including only participants with mild TBI).

## Data Availability

Owing to the sensitivity of psychiatric patient data, our IRB requires individualized review before data sharing. We have produced anonymized data related to the present findings for sharing with all scientists with research and data safeguarding plans that comport with Stanford University guidelines. Please contact N. Williams at nolanw@stanford.edu with data-sharing requests.
