# Supplementary material for: Magnesium–ibogaine therapy in veterans with traumatic brain injuries
Source: Nat Med. 2024 Jan 5;30(2):373–81. doi: 10.1038/s41591-023-02705-w (PMC10878970; doi:10.1038/s41591-023-02705-w)
Supplement: Supplementary file 1 — Reporting Summary [file 41591_2023_2705_MOESM1_ESM.pdf]

nature portfolio

Corresponding author(s): Nolan Williams

Last updated by author(s): Nov 2, 2023

Reporting Summary

Nature Portfolio wishes to improve the reproducibility of the work that we publish. This form provides structure for consistency and transparency in reporting. For further information on Nature Portfolio policies, see our Editorial Policies and the Editorial Policy Checklist.

Statistics

For all statistical analyses, confirm that the following items are present in the figure legend, table legend, main text, or Methods section.

n/a	Confirmed	
☐	☒	The exact sample size (*n*) for each experimental group/condition, given as a discrete number and unit of measurement
☐	☒	A statement on whether measurements were taken from distinct samples or whether the same sample was measured repeatedly
☐	☒	The statistical test(s) used AND whether they are one- or two-sided<br>*Only common tests should be described solely by name; describe more complex techniques in the Methods section.*
☐	☒	A description of all covariates tested
☐	☒	A description of any assumptions or corrections, such as tests of normality and adjustment for multiple comparisons
☐	☒	A full description of the statistical parameters including central tendency (e.g. means) or other basic estimates (e.g. regression coefficient) AND variation (e.g. standard deviation) or associated estimates of uncertainty (e.g. confidence intervals)
☐	☒	For null hypothesis testing, the test statistic (e.g. *F*, *t*, *r*) with confidence intervals, effect sizes, degrees of freedom and *P* value noted<br>*Give P values as exact values whenever suitable.*
☒	☐	For Bayesian analysis, information on the choice of priors and Markov chain Monte Carlo settings
☒	☐	For hierarchical and complex designs, identification of the appropriate level for tests and full reporting of outcomes
☐	☒	Estimates of effect sizes (e.g. Cohen's *d*, Pearson's *r*), indicating how they were calculated

Our web collection on statistics for biologists contains articles on many of the points above.

Software and code

Policy information about availability of computer code

Data collection	Data was collected in REDCap
Data analysis	All Statistical Analyses were performed in MATLAB R2021a. Figures were created using Excel 365.

For manuscripts utilizing custom algorithms or software that are central to the research but not yet described in published literature, software must be made available to editors and reviewers. We strongly encourage code deposition in a community repository (e.g. GitHub). See the Nature Portfolio guidelines for submitting code & software for further information.

Data

Policy information about availability of data

All manuscripts must include a data availability statement. This statement should provide the following information, where applicable:

- Accession codes, unique identifiers, or web links for publicly available datasets
- A description of any restrictions on data availability
- For clinical datasets or third party data, please ensure that the statement adheres to our policy

Due to the sensitivity of psychiatric patient data, our institutional review board requires individualized review prior to data sharing. We have produced anonymized data related to the present findings for sharing with all scientists with research and data safeguarding plans that comport with Stanford University guidelines. Please contact Dr. Nolan Williams at nolanw@stanford.edu with data-sharing requests

Research involving human participants, their data, or biological material

Policy information about studies with human participants or human data. See also policy information about sex, gender (identity/presentation), and sexual orientation and race, ethnicity and racism.

Reporting on sex and gender	All participants self identified as male. Gender or biological sex was not used for analysis purposes. Gender was determined by the participants using classification terms provided by the researchers. Classification terms were: "male", "female", or "other".
Reporting on race, ethnicity, or other socially relevant groupings	Reported in Table 1
Population characteristics	Reported in Table 1
Recruitment	Participants were referred to the study by VETS, Inc. after being approved for a treatment grant.<br>Veterans who were assessed by VETS as requiring treatment acutely were not referred to the study. As described in the online methods, participants were SOV who had independently scheduled themselves for MISTIC. As detailed in the discussion section, the study was not controlled, and so the relative contribution of this potential bias to the therapeutic response cannot be determined.
Ethics oversight	All research procedures were approved by Stanford University Institutional Review Board.

Note that full information on the approval of the study protocol must also be provided in the manuscript.

Field-specific reporting

Please select the one below that is the best fit for your research. If you are not sure, read the appropriate sections before making your selection.

☐ Life sciences ☒ Behavioural & social sciences ☐ Ecological, evolutionary & environmental sciences

For a reference copy of the document with all sections, see nature.com/documents/nr-reporting-summary-flat.pdf

Behavioural & social sciences study design

All studies must disclose on these points even when the disclosure is negative.

Study description	Pilot study This study evaluating the safety and efficacy of Ibogaine-Magnesium therapy in treating functional disability and crossdiagnostic psychological impairments in Veterans suffering from Traumatic Brain Injury (TBI) caused by repeated combat blast exposure. Analysis was quantitative.
Research sample	Study population consisted of 30 US special operations veterans (aged 18-70; all males) with a history of traumatic brain injury. The study sample is representative of Special Operation Veterans (SOV). SOV was selected as the target population considering the high burden and prevalence of TBI.
Sampling strategy	Clinician administered scales were collected by a neuropsychologist and logged in REDcap. Self report scales were independently logged by the participant using a REDcap instrument. As an observational study, no power calculation was performed. Sample size of 30 was selected to balance our desire for a larger sample with the importance of providing prompt preliminary safety and efficacy data to other SOV who are considering this treatment given their potentially vulnerable status. Sample size of 30 was selected to balance our desire for a larger sample with the importance of providing prompt preliminary safety and efficacy data to other SOV who are considering this treatment given their potentially vulnerable status.
Data collection	Recruitment took place between November 2021 to September 2022. Clinical interviews were conducted via Zoom or in person at Stanford University. Neuropsychological assessments were conducted in person at Stanford University.<br>Clinician administered scales were collected by a neuropsychologist and logged in REDcap. Self report scales were independently logged by the participant using a REDcap instrument. Participants were assessed individually by research staff, and assessments were video recorded with participant consent. Clinician-administered scales were collected by a neuropsychologist and logged in REDCap. Self-report scales were independently logged by the participant using a REDCap instrument. As the study was open-label, the researchers were not blinded to experimental conditions or study hypotheses.
Timing	November 2021 to September 2022
Data exclusions	No enrolled participants were excluded from analysis.
Non-participation	3 Participants did not meet inclusion criteria.
Randomization	The trial was not randomized. Age combat exposure score, and total number of TBIs were controlled for by adding them as random effects in LME models.

Reporting for specific materials, systems and methods

We require information from authors about some types of materials, experimental systems and methods used in many studies. Here, indicate whether each material, system or method listed is relevant to your study. If you are not sure if a list item applies to your research, read the appropriate section before selecting a response.

Materials & experimental systems

n/a	Involved in the study
☒	☐ Antibodies
☒	☐ Eukaryotic cell lines
☒	☐ Palaeontology and archaeology
☒	☐ Animals and other organisms
☐	☒ Clinical data
☒	☐ Dual use research of concern
☒	☐ Plants

Methods

n/a	Involved in the study
☒	☐ ChIP-seq
☒	☐ Flow cytometry
☒	☐ MRI-based neuroimaging

Clinical data

Policy information about clinical studies

All manuscripts should comply with the ICMJE guidelines for publication of clinical research and a completed CONSORT checklist must be included with all submissions.

Clinical trial registration	NCT04313712
Study protocol	Study protocol was submitted to nature medicine along with all manuscript materials.
Data collection	Data was collected at Stanford Stanford University as well as remotely via secure virtual platform between November 2021 to September 2022.
Outcomes	The pre-specified primary outcome was change in the World Health Organization Disability Assessment Schedule 2.0 (WHODAS) from baseline to post-treatment, with change from baseline to the one-month follow-up a secondary outcome. Additional pre-specified secondary outcomes included post-treatment changes on the Clinician Administered PTSD Scale (CAPS-5), Montgomery-Åsberg Depression Rating Scale (MADRS), the Hamilton-Anxiety Rating Scale (HAM-A), and neuropsychological testing. To assess the significance of post-treatment changes, linear mixed effects (LME) models were used for each outcome measure. False Discovery Rate (FDR)23 was applied to correct for multiple comparisons. All statistical analyses were performed in MATLAB R2021a. Figures were created using Excel 365. LME models were used for each outcome measure (WHODAS, CAPS-5, MADRS, and HAM-A). Specifically, outcome measure scores served as the dependent variable and time point (baseline, post-MISTIC, one-month follow-up) as the independent variable, with a fixed slope and random intercept; age, combat exposure (measured by the CES), and total number of TBIs were included in the model as random effects.
